# The complete mitochondrial genome of *Chaetodon wiebeli* (Chaetodontiformes, Chaetodontidae)

**DOI:** 10.1080/23802359.2019.1667894

**Published:** 2019-09-20

**Authors:** Yang Yukai, Huang Xiaolin, Lin Heizhao, Li Tao, Yu Wei, Huang Zhong

**Affiliations:** a Key Lab of South China Sea Fishery Resources Exploitation and Utilization, Ministry of Agriculture and Rural Affairs, South China Sea Fisheries Research Institute, Chinese Academy of Fishery Sciences, Guangzhou, Guangdong, China;; b Shenzhen Base of South China Sea Fisheries Research Institute, Chinese Academy of Fishery Sciences, Shenzhen, Guangdong, China

**Keywords:** *Chaetodon wiebeli*, Chaetodontidae;·mitochondrial genome

## Abstract

*Chaetodon wiebeli* is one of the most important genera of Chaetodontidae. However, the systemic classification and taxonomic studies have so far been limited. In this study, we report the complete mitochondrial genome sequence of *C. wiebeli*. The mitogenome has 16,523 bp (54.3% A + T content) and is made up of a total of 37 genes (13 protein-coding, 22 transfer RNAs, and 2 ribosomal RNAs), and a putative control region. This study will provide useful genetic information for future phylogenetic and taxonomic classification of Chaetodontidae.

*Chaetodon wiebeli* belongs to the family Chaetodontidae and the order Chaetodontiformes, and is distributed in Western Pacific: Japan to Thailand; including the Ryukyu Islands, Taiwan, the South China Sea, and the Gulf of Thailand (Froese and Pauly 2019).

There is no report of the complete genome of this species *C. wiebeli*, which was developed in Shenzhen, Guangdong Province, Republic of China (N22°37′34″, E114°41′06″) in October 2018. Therefore, it is very important to characterize the complete mitogenome of this species, which can be utilized in research on the taxonomic resolution, population genetic structure and phylogeography, and phylogenetic relationship. Total DNA was extracted from muscle following TIANamp Marine Animals DNA Kit (Tiangen, China), and NOVOPlasty software was used to assemble the mitogenomes, the mistake parameter was set by default (Dierckxsens et al. 2017). The samples were stored at −80 °C in Key Lab of South China Sea Fishery Resources Exploitation and Utilization, Ministry of Agriculture and Rural Affairs, South China Sea Fisheries Research Institute, Chinese Academy of Fishery Sciences, Guangzhou, China. The number is CW-1.

In this study, we obtained the complete mitochondrial genome of *C. wiebeli*. Its mitochondrial genome has been deposited in the GenBank under accession number MN170515. For a better understanding of the genetic status and the evolutionary study, we focused on the genetic information contained in the complete mitochondrial genomes of the fish.

The complete mitogenome of the *C. wiebeli* was 16,523 bp in length. The genomic organization was identical to those of typical vertebrate mitochondrial genomes, including two rRNA genes, 13 protein-coding genes, 22 tRNA genes, a light-strand replication origin (OL), and a putative control region (CR). The overall base composition was 27.9% of A, 26.4% of T, 29.1% of C, and 16.6% of G with a slight A + T bias (54.3%) like other vertebrate mitochondrial genomes. The features mentioned above were in accordance with typical Chaetodontidae fish mitogenome.

For the 13 protein-coding genes, 12 genes started with ATG while only *COI* started with GTG. Five genes shared the termination codon TAA (*COI*, *ATPase8*, *ND1*, *ND4L*, and *ND6*), one with TAG (*ND5*), the remaining had incomplete stop codons (*COII*, *COIII*, *ND2*, *ND3*, *ND4*, *ATPase6*, and *Cytb*). This feature was common among vertebrate mitochondrial protein-coding genes. *Chaetodon wiebeli* had two non-coding regions, the L-strand replication origin region (36 bp) located between tRNA-Asn and tRNA-Cys, and the control region (860 bp) located within the tRNA-Pro and tRNA-Phe. Except for eight tRNA (tRNA-Ser, tRNA-Pro, tRNA-Glu, tRNA-Tyr, tRNA-Cys, tRNA-Asn, tRNA-Ala, and tRNA-Gln) and the *ND6* gene, which were coded on the L-strand, the others were encoded on the H-strand. This feature is similar to other fish mitochondrial genes. The complete mitogenome sequence had 16s RNA (1693 bp) and 12s RNA (949 bp), which were located between tRNA-Phe and tRNA-Leu and separated by *tRNA-Val* gene. The location is same with most vertebrates that have high conservation.

To determine the taxonomic status of *C. wiebeli*, we reconstructed the phylogeny of this species with other natural populations based on the *COI* gene, *Salvelinus malma* as a foreign group (Yang et al. 2017). The phylogenetic tree showed that *C. wiebeli* has a closer relationship with *Chaetodon auripes* (Figure 1). The phylogeny was reconstructed based on the General Time Reversible + Invariant + gamma sites (GTR + I + G) model of nucleotide substitution using Mega7 (Kumar et al. 2016). The complete mitochondrial genome sequence of the *C. wiebeli* provided an important dataset for a better understanding of the mitogenomic diversities and evolution in fish, as well as, novel genetic markers for studying population genetics and species identification.

**Figure 1.**
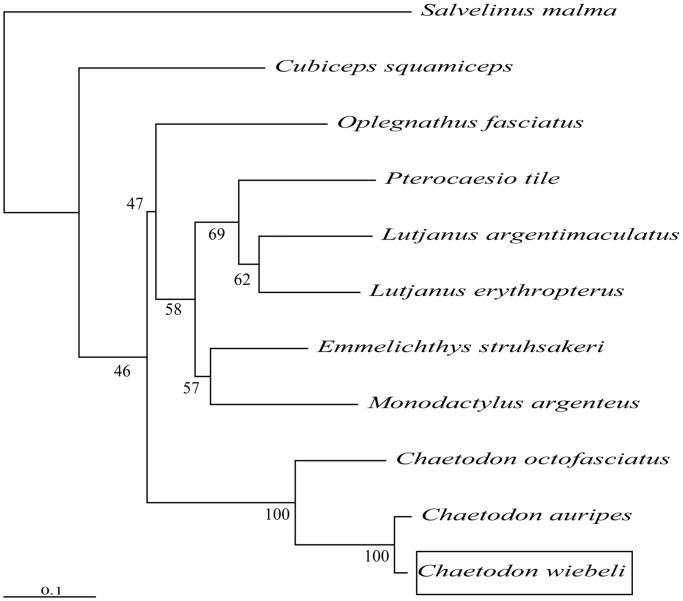
The phylogenetic relationship was estimated using the maximum-likelihood method for the *COI* genes. Genbank accession Numbers: *Chaetodon auripes* (AP006004), *Chaetodon octofasciatus* (NC_040865), *Oplegnathus fasciatus* (AP006010), *Emmelichthys struhsakeri* (AP004446), *Pterocaesio tile* (AP004447), *Lutjanus erythropterus* (KP939271), *Monodactylus argenteus* (AP009169), *Cubiceps squamiceps* (AB205443), *Lutjanus argentimaculatus* (JN182927), *Salvelinus malma* (MF680544), and *Chaetodon wiebeli* (MN170515). The numbers at the nodes are bootstrap percent probability values based on 1000 replications.
